# Projected climate change impacts on the phylogenetic diversity of the world's terrestrial birds: more than species numbers

**DOI:** 10.1098/rspb.2021.2184

**Published:** 2022-07-27

**Authors:** Alke Voskamp, Christian Hof, Matthias F. Biber, Katrin Böhning-Gaese, Thomas Hickler, Aidin Niamir, Stephen G. Willis, Susanne A. Fritz

**Affiliations:** ^1^ Senckenberg Biodiversity and Climate Research Centre, 60325 Frankfurt, Germany; ^2^ Terrestrial Ecology Research Group, Technical University of Munich, 85354 Freising, Germany; ^3^ Institute of Physical Geography, Goethe University, 60438 Frankfurt, Germany; ^4^ Department of Biosciences, Durham University, DH1 3LE Durham, UK; ^5^ Department of Biological Sciences, Goethe University Frankfurt, 60438 Frankfurt, Germany; ^6^ Institut für Geowissenschaften, Goethe University Frankfurt, 60438 Frankfurt, Germany

**Keywords:** phylogenetic assemblage structure, species range shifts, ecological forecasting, ISIMIP

## Abstract

Ongoing climate change is a major threat to biodiversity. As abiotic tolerances and dispersal abilities vary, species-specific responses have the potential to further amplify or ameliorate the ensuing impacts on species assemblages. Here, we investigate the effects of climate change on species distributions across non-marine birds, quantifying its projected impact on species richness (SR) as well as on different aspects of phylogenetic diversity globally. Going beyond previous work, we disentangle the potential impacts of species gains versus losses on assemblage-level phylogenetic diversity under climate change and compare the projected impacts to randomized assemblage changes. We show that beyond its effects on SR, climate change could have profound impacts on assemblage-level phylogenetic diversity and composition, which differ significantly from random changes and among regions. Though marked species losses are most frequent in tropical and subtropical areas in our projections, phylogenetic restructuring of species communities is likely to occur all across the globe. Furthermore, our results indicate that the most severe changes to the phylogenetic diversity of local assemblages are likely to be caused by species range shifts and local species gains rather than range reductions and extinctions. Our findings highlight the importance of considering diverse measures in climate impact assessments.

## 1. Introduction

Global warming has been identified as one of five main anthropogenic drivers of global biodiversity loss [1], and it is projected to increasingly threaten biodiversity in the future [2,3]. First responses of species to climate change have already been reported [4], indicating the three possible ways in which species can adapt to global warming (i.e. through shifts in phenology, physiology or geographic ranges [5]). In particular, changes in species abundance and distribution have already been observed [6], with stronger changes towards higher latitudes and elevations [6]. Idiosyncratic range shifts with species-specific directions and extent have also been recorded [7] and have the potential to be especially problematic, since they will probably result in a reshuffling of species assemblages. Potential direct and indirect consequences of such reshuffling could include changes to the competitive balance between species within these assemblages [8] and altered predator–prey relationships [9], as well as changes to the trait composition of local assemblages [10] and subsequently the provision of ecological functions and services [11].

Changes in the structure of species assemblages, caused by immigration or extinction, indicate potential changes in ecological processes. These changes can be assessed through phylogenetic diversity, i.e. the diversity of phylogenetic lineages present in the assemblage [12]. One aspect of phylogenetic diversity is the total evolutionary diversity of a species assemblage, which is frequently calculated as the sum of all branch lengths in a phylogenetic tree that spans a set of species (Faith's phylogenetic diversity, named Faith PD hereafter, and assumed to represent total standing feature or trait diversity [13]). A strong reduction in the trait diversity of a species assemblage might reduce the possibilities of an assemblage to respond to future changes. As an alternative metric, the phylogenetic relatedness of the species assemblage can be assessed using the mean pairwise distance (MPD), which is the inverse of the average relatedness between all species pairs [14,15]. Under the assumption that closely related species have a tendency to share more similar functional traits than very distantly related species [16], an increase in the relatedness of species within an assemblage could imply a reduction in the diversity of traits present (but see [17,18]), potentially also increasing the vulnerability of the assemblage towards environmental change.

Comparing these two metrics of phylogenetic diversity when investigating temporal change in species assemblages can yield valuable information on the underlying compositional changes that are taking place. Though both metrics measure related aspects of phylogenetic diversity, they are mathematically independent and react differently to changes in species assemblages, allowing inference of four potential ways in which the phylogenetic structure of these assemblages could shift when undergoing climate-induced compositional changes as follows: (i) both MPD and Faith PD could decrease, which indicates a pattern of increasing phylogenetic homogenization (e.g. through a loss of species or phylogenetic lineages that were not closely related to remaining lineages); (ii) both MPD and Faith PD could increase, indicating increasing phylogenetic diversification (e.g. through a gain of phylogenetically unique species that decrease average relatedness); (iii) MPD could increase while Faith PD decreases, indicating increasing phylogenetic over-dispersion (e.g. through a loss of closely related species); or (iv) MPD could decrease while Faith PD increases, indicating increasing phylogenetic clustering of the species assemblage (e.g. through a gain of closely related species or evolutionary lineages).

Regardless of direction, changes in species richness (SR) (i.e. the gain and loss of species into and from a species assemblage) could result in severe changes to the phylogenetic structure of species assemblages that are different from expectations under phylogenetically random species gain or loss and likely to have strong ecological effects. To date, the phylogenetic signal of species extinctions through climate change has been found to be rather weak across larger scales (e.g. the tree of life for Europe for plants, mammals and birds [37]). However, there is mixed evidence for strong phylogenetic impacts at the local scale, where climate change might result in the loss of entire clades or distinct evolutionary lineages from assemblages [19–21], causing phylogenetic homogenization. It is therefore unclear to what extent such findings hold true for other taxa and more broadly across the world.

An additional complication that has been largely ignored in existing projections of future climate change impact is that local phylogenetic diversity will not only be subject to change through species losses but also through species that newly arrive into an area due to shifts in species geographic range boundaries. Disentangling the impacts of species losses and gains, rather than looking at the overall change in phylogenetic diversity, is important to fully assess and understand the projected changes. For example, a given assemblage could decrease significantly in phylogenetic diversity through species loss, but also increase significantly through species immigration, so the overall change in phylogenetic diversity under climate change would be marginal despite a significant change in the underlying phylogenetic structure of the assemblage.

Here, we first (i) investigate how projected range shifts and (local) species extinctions induced by climate change affect the spatial pattern of phylogenetic diversity for an entire taxon, using the world's terrestrial bird species. Second, (ii) we compare how the projected changes in local species assemblages differ from what would be expected at random given the projected local SR change and disentangle non-random changes through local species gain and loss.

Faith PD has often been found to be highly correlated to SR for various taxa [22,23], with some local exceptions where the correlation is less strong [24,25]. By contrast, MPD is independent of SR [23,24]. Therefore, we expect (i) projected changes in Faith PD to largely reflect the changes in SR, whereas projected changes in MPD should be decoupled especially in regions where uniform range shifts occur in some but not all avian taxa, as might be the case across the northern temperate and boreal latitudes. Further, we expect (ii) changes in phylogenetic diversity through projected species loss to be decoupled from those caused by projected species gain, if phylogenetically different species are projected to be lost versus gained locally.

## 2. Material and methods

Preparation of species distribution and climatic data, as well as the format for species distribution models (SDMs) and the design of the chosen dispersal buffer, follow methods described in Hof *et al*. [26]. Here, we provide a brief summary, with full details in the supplementary material. The extent of our study is global, covering all terrestrial areas excluding Antarctica, and grain sizes of analyses were cells of 0.5° latitude/longitude.

### (a) Species distributions, projections and phylogenetic data

We used expert range maps for the breeding ranges of 9882 terrestrial bird species from BirdLife International [27], considering only species with ranges of greater than 10 grid cells (electronic supplementary material, figure S1) for the modelling. We used the bias-corrected climate data from the Coupled Model Intercomparison Project Phase 5 (CMIP5), provided by the Inter-Sectoral Model Intercomparison Project (ISIMIP), considering two different representative concentration pathways (RCPs) RCP2.6 and RCP6.0 as well as four different GCMs (GFDL-ESM2M, HadGEM2-ES, IPSL-CM5A-LR and MIROC5) [28]. Furthermore, we applied two different SDM algorithms, generalized additive models [29,30] and generalized boosted regression models [31], to derive the relationship between a species' current range extent and the bioclimatic variables. We used ensemble projections across the four GCMs and the two SDMs (see electronic supplementary material, figure S2 for the uncertainty around the projected SR values) to generate current and future species presence–absence matrices for the years 1995 and 2080 under the two RCPs for different dispersal scenarios (low and medium dispersal, results presented are based on the latter), following the methods of Hof *et al*. [26].

For the phylogenetic analysis, we compiled a consensus tree based on a full species-level phylogeny of extant birds [32]. We found a very low level of variation in PD when using phylogenetic trees that were randomly sampled from the pseudo-posterior distribution rather than a consensus phylogeny. We matched the taxonomy of the phylogeny [32] with that of the range maps [27], resolving all conflicting species, which resulted in a final combined dataset for 8768 species.

### (b) Projected spatial patterns in phylogenetic diversity metrics

To extract potential changes in SR, Faith PD and MPD, we derived current (electronic supplementary material, figure S3) and future species assemblages for each grid cell globally based on the projected species distributions. Then, *change in SR* was calculated as the proportional change between the number of species projected to occur in a grid cell currently and in the future. Second, total *change in Faith PD* was calculated as the proportional change between the Faith PD (calculated following [13]) values for the species projected to occur in a grid cell in present and future time periods. Finally, total *change in MPD* was calculated as the proportional change between the current and future MPD (calculated following [15]) value of a grid cell.

### (c) Projected non-random changes in phylogenetic assemblage structure

**Figure 1.**
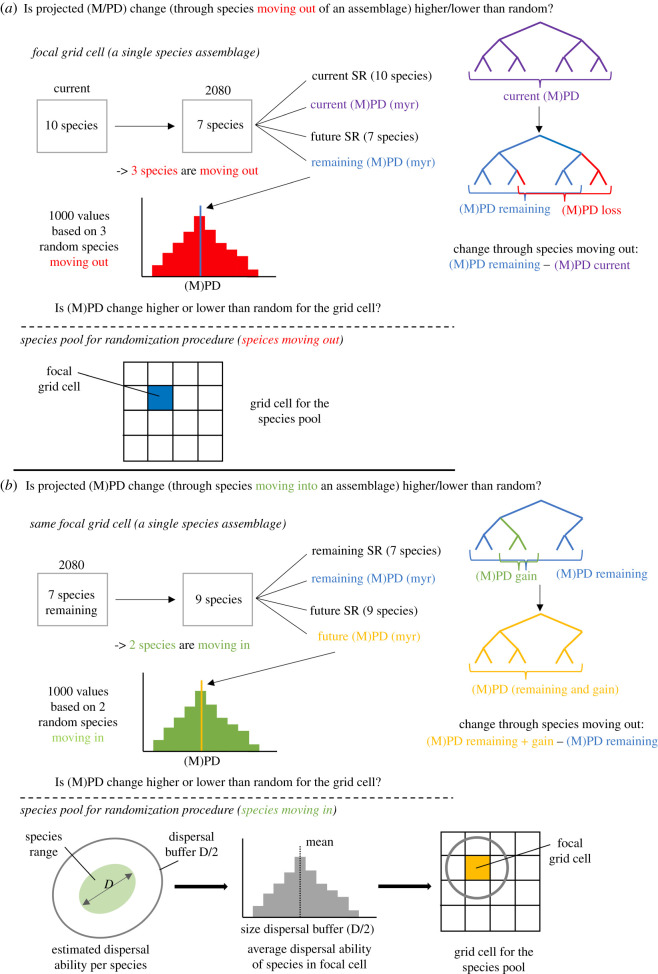
Comparison of the projected changes of a species assemblage (grid cell) in phylogenetic diversity metrics, Faith PD (called PD in the flow diagram) and MPD, based on species that are projected to be lost from (*a*) and gained into (*b*) the assemblage, with the expected changes in metrics based on the same number of species being lost and gained at random. In this example, we assume that (*a*) there were 10 species in the assemblage initially and we project seven species to remain in the assemblage (with three species projected to emigrate or go extinct). To calculate the expectation for random species loss, we then drop three random species from the list of 10 species 1000 times; the species pool is the focal assemblage. Then, we assume that (*b*) seven species remain (we use the remaining species here to keep the changes by species lost and gained comparable) in the assemblage and two species are projected to be gained. To calculate the expectation for random species gain, we draw two random species from the species pool 1000 times, where the pool consists of candidate species that occur within a colonizable distance of the focal assemblage. We estimated this distance as the mean dispersal ability across the species in the focal grid cell and approximated species' dispersal ability as half the value of their longest range diameter (D). (Online version in colour.)

We decomposed the net change in SR in a given assemblage (grid cell) into the species persisting, the species projected to be lost (through extinction or emigration), and the species projected to be gained (through immigration) under climate change (figure 1). We then also decomposed changes in the phylogenetic metrics into those caused by species loss versus those caused by species gain (figure 1). Changes caused by species loss were calculated as Faith PD_remaining_ *minus* Faith PD_current_, or MPD_remaining_ *minus* MPD_current_, respectively (figure 1*a*); changes caused by species gain were calculated as Faith PD_(remaining+gain)_ *minus* Faith PD_remaining_, or MPD_(remaining+gain)_ *minus* MPD_remaining_, respectively (figure 1*b*). We evaluated whether the projected changes in Faith PD and MPD were different from what could be expected if the species that moved in or out of an area were randomly distributed across the phylogeny. These randomizations constitute a widely accepted null model approach and are necessary because the structure of the phylogeny determines the possible extent of projected changes in Faith PD and MPD given a particular species assemblage and number of species moving in or out [33,34].

Random changes through species loss were calculated in both phylogenetic metrics of a species assemblage (grid cell) using the list of species projected to be currently present in the assemblage by the SDMs. The same number of species as projected to be lost from the assemblage was then repeatedly (1000 times) removed from the current species assemblage at random, and both phylogenetic metrics were recalculated each time (figure 1*a*). To assess the statistical significance of differences between the projected change in the phylogenetic metrics and changes based on random species removals, we calculated a two-sided *p*-value as the proportion of the 1000 random values that were smaller or larger than the observed value.

We followed an equivalent approach to assess non-randomness of the change in both phylogenetic metrics based on species projected to be gained by the assemblage (immigrating into the assemblage; figure 1*b*). We extracted the number of species that were projected to be gained by the assemblage (grid cell) and then, using this number, we randomly added new species to those that were projected to remain in the assemblage under climate change, using a species pool based on estimated species’ dispersal abilities. This species pool definition means that our null model for species gain is more biologically defined than the null model for species loss (figure 1*b*; see electronic supplementary material for a detailed description of the species pool and the null models). Again, we calculated a two-sided *p*-value indicating if there was a significant difference between the projected change in both phylogenetic metrics and changes based on random species being gained by the assemblage.

All data and codes needed to re-run the analysis and create the plots can be found on Dryad (doi:10.5061/dryad.cjsxksn6j). All analyses were performed in R [35].

## 3. Results

We present results for climate change projections in 2080 given a medium emission scenario and assuming medium species-level dispersal ability. All patterns were consistent when re-running analyses for a low emission scenario (electronic supplementary material, figure S4–S6 and tables S4–S5) and a different dispersal scenario (electronic supplementary material, figure S7–S9 and tables S6–S7).

### (a) Projected spatial patterns in phylogenetic diversity metrics

**Figure 2.**
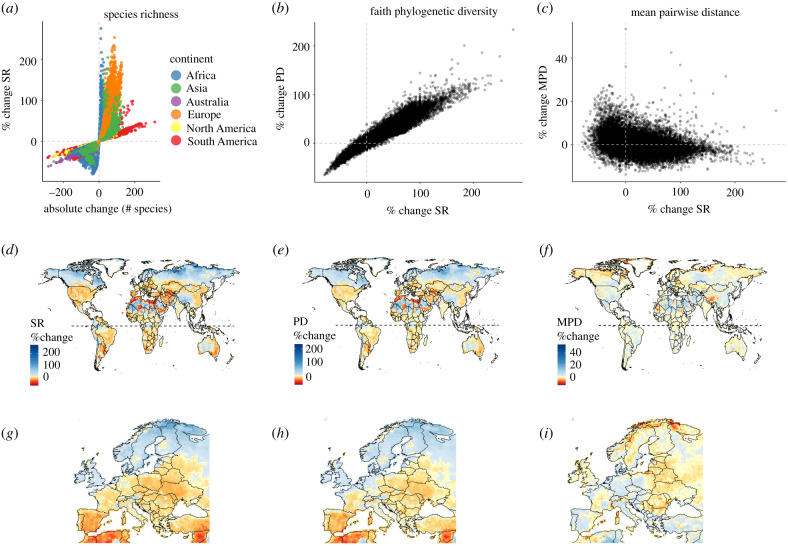
Projected changes in SR, Faith's phylogenetic diversity (Faith PD) and MPD under a medium emission scenario (RCP6.0) and a medium dispersal scenario by 2080; (*a*) shows the percentage change in SR against absolute change in SR; (*b*) the percentage change in Faith PD against percentage change in SR; (*c*) the percentage change in MPD against percentage change in SR; (*d*–*f*) the spatial distribution of percentage changes in (*d*) SR, (*e*) Faith PD and (*f*) MPD. Spatial distributions for all three measures are shown in enlarged insets for Europe (*g*–*i*). Red indicates a negative change (e.g. loss in SR, Faith PD or MPD); blue indicates a positive change (e.g. gain in SR, Faith PD or MPD). (Online version in colour.)

The projected changes in SR within assemblages (grid cells) do not differ greatly across continents but do differ within them (figure 2*a*). These SR changes are spatially highly correlated with projected total changes in Faith PD across the globe (figure 2*b*). Although proportional decreases in both SR and Faith PD are likely to be most extreme in species-poor regions (e.g. the Saharan and Arabian deserts), we also project high proportional decreases in some of the species-rich regions of the world, e.g. in parts of South America and on New Guinea (figure 2*d,e*). Whereas assemblages with decreases up to 30% in both metrics may be found across all continents, those with a projected proportional gain are especially widespread at high northern latitudes across the Nearctic and Palaearctic realm. The projected total changes in MPD (figure 2*f*) differ substantially from the projected changes in SR (figure 2*c*) and Faith PD, and are often the opposite to these, which is especially apparent in Europe (figure 2*g–i*). The areas with strong increases in SR and PD across the northern Nearctic and Palaearctic are projected to decrease in MPD, whereas assemblages with SR and PD increases in South America and New Guinea are projected to also increase in MPD.

**Figure 3.**
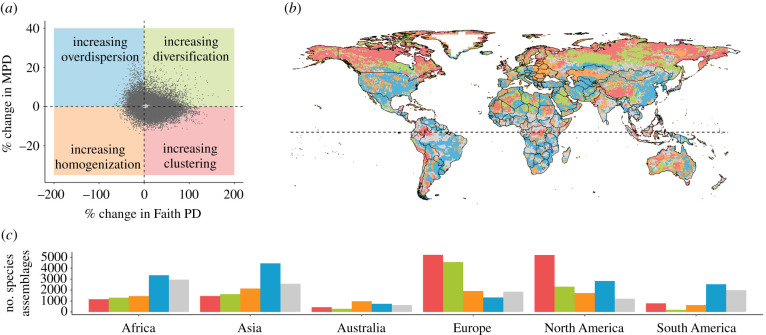
Comparison of the direction of projected changes in phylogenetic assemblage structure as indicated by MPD versus by Faith's phylogenetic diversity (Faith PD) under a medium emission scenario (RCP 6.0) assuming a medium dispersal scenario by 2080. The scatterplot (*a*) shows percentage change in MPD against percentage change in Faith PD, divided into four categories of change using the median along each axis. The map (*b*) shows the spatial distribution of the species assemblages falling into one of these four categories, and the bar chart (*c*) shows the number of assemblages per category across different continents. The four defined categories are grid cells with a projected gain in MPD and loss in Faith PD leading to increasing phylogenetic over-dispersion of these species assemblages (blue); grid cells with a projected loss in both MPD and Faith PD, leading to increasing phylogenetic homogenization (yellow); grid cells with a projected loss of MPD and gain in Faith PD, indicating increasing phylogenetic clustering (red) and grid cells with a projected gain in both MPD and Faith PD, indicating increasing phylogenetic diversification (green). Grey indicates the assemblages in the lowest quartile of combined changes, which were considered to not undergo a significant change in any direction. (Online version in colour.)

When directly comparing projected changes in the two phylogenetic structure metrics and disregarding the quartile of assemblages with least change along both metrics (figure 3), we find that Faith PD and MPD are projected to change into opposite directions in approximately 48% of those assemblages (electronic supplementary material, table S2 and discussion). Half of these assemblages (i.e. 29% globally) are projected to experience an increase in MPD and a decrease in Faith PD (i.e. a decrease in average relatedness and in standing evolutionary history), leading to increasing phylogenetic over-dispersion of these species assemblages (figure 3; electronic supplementary material, table S2). Further, 19% of the species assemblages are projected to experience the opposite (decreasing MPD and increasing Faith PD), indicating increasing relatedness and evolutionary history and therefore phylogenetic clustering. Projected changes of both metrics in the same direction were slightly rarer in comparison (decreases in both MPD and Faith PD (i.e. increasing homogenization) in 16% of the species assemblages globally, and increases in both in 14%, indicating increasing diversification). Whereas assemblages with a projected increase in phylogenetic clustering dominated in Europe and North America, those with increasing over-dispersion were most frequent in Asia, Africa and South America (figure 3; electronic supplementary material, table S2).

### (b) Projected non-random changes in phylogenetic assemblage structure

**Figure 4.**
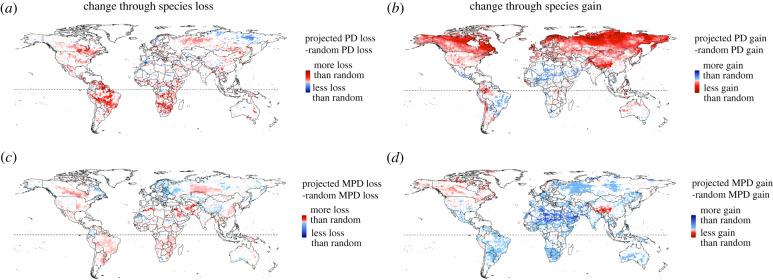
The significance and direction of projected changes in Faith's phylogenetic diversity (Faith PD) and MPD of species assemblages (grid cells), through species that are projected to be lost from (*a*,*c*) and gained into (*b*,*d*) assemblages, in comparison to expected changes if species were lost and gained at random. Difference values for species being lost from an assemblage are calculated as shown in figure 1. For the maps of change in Faith PD/MPD through species being lost from an assemblage (*a*,*c*), red indicates that the loss of Faith PD/MPD caused by the species that are projected to be lost from the assemblage is significantly higher than what would be expected if the same number of random species would be lost; blue indicates that the loss is significantly lower than what would be expected if random species would be lost (significance is derived using a two-sided *p*-value less than 0.05 or greater than 0.95). For the maps of change in Faith PD/MPD through species being gained into an assemblage (*b,d*), red indicates that the gain in Faith PD/MPD through the species projected to be gained into the assemblage is significantly lower than what would be expected if the same number of random species would be gained into the assemblage, blue indicates that the gain is significantly higher than what would be expected if random species would be gained. A gain or loss in Faith PD signifies a significant increase or decrease in total evolutionary history represented, respectively; a gain or loss in MPD signifies a significant decrease or increase in average relatedness, respectively. White areas in each map have no significant changes compared to random species gain or loss. Results are shown for a medium emission scenario (RCP6.0) and a medium dispersal scenario by 2080. (Online version in colour.)

When we teased apart whether changes in phylogenetic assemblage structure from species loss versus species gain were statistically significant, we found differences among PD and MPD results in many areas that confirmed the pattern comparison described above. Small areas exist on all continents where the decrease in Faith PD through a projected species loss is significantly less severe than expected from randomized species moving out of the assemblage; these areas are most frequent in the northern Palaearctic and Nearctic (figure 4*a*, blue areas). These assemblages are projected to lose species through climate change that represent unusually low amounts of evolutionary history, but these species losses apparently do not lead to consistent significant changes in net relatedness of those assemblages (i.e. in MPD, figure 4*c*). By contrast, areas with a significantly stronger decrease in Faith PD than we would expect at random (through the loss of species), are most common in central South America and southern African regions, but also prevail across parts of North America and northern Asia (figure 4*a*, red areas). The assemblages in these areas are projected to lose species that represent disproportionately high amounts of evolutionary history. They also mostly experience parallel significant decreases in MPD compared to random species loss (figure 4*c*, red areas), indicating significant increases in net relatedness that further highlight the losses of evolutionary unique species we find in Faith PD analyses.

Focusing on species gains, we project those areas with significantly lower increases in Faith PD than expected to be most frequent at high northern latitudes, stretching across the entire Nearctic and Palaearctic realm, but also across parts of South America and Australia (figure 4*b*, red areas). This category of significantly lower increase in Faith PD is the most widespread in extent, indicating that not only are more areas projected to gain more species than lose them (figure 2*d*), but also that the species gains in these areas may not lead to the expected increases in evolutionary history. Similarly, the significant changes in MPD from species gains are more prevalent across the globe than from species loss, but the predominant pattern is that of unexpectedly strong increases in MPD (figure 4*d*, blue areas), indicating that overall relatedness often decreases significantly through species projected to be gained under climate change. In addition, in those areas where the projected changes through species gain in Faith PD and in MPD are significant, these changes are often in opposite directions (e.g. compare red areas in figure 4*b*,*d*). We provide a more in-depth description of MPD results and their comparison to Faith PD patterns in the supplement.

When comparing the significant non-random changes from species losses versus gains within assemblages within each phylogenetic diversity metric, we project that there are numerous assemblages with overlap (electronic supplementary material, table S3). Projected future changes for each phylogenetic diversity metric, Faith PD and MPD, overlap most in assemblages that may lose more phylogenetic diversity than if species were lost randomly and simultaneously gain more phylogenetic diversity than if species were gained randomly (greater than 14% of global assemblages; electronic supplementary material, table S3). This indicates that particularly high proportions of assemblages in most continents (especially in South America, Africa and North America) are projected to experience major species reshuffling through both species losses and gains (see electronic supplementary material, table S3 for more discussion).

## 4. Discussion

We find that including different measures of phylogenetic diversity (Faith PD and MPD) in future climate change impact assessments can help to identify species assemblages that might be under higher threat than projected changes in local SR alone would suggest. The projected impacts of climate change not only affect the amount of evolutionary history stored within species assemblages but might also have significant impact on the phylogenetic structure of species communities, which plays an important role in shaping ecological processes and ecosystem functioning because phylogenetic composition is usually linked to the composition in functional traits [36]. Though marked species losses are projected to be most frequent in tropical and subtropical areas, phylogenetic restructuring of species communities is projected to occur all across the globe.

Projected changes through species gain were notably stronger than those through species loss across the northern latitudes especially, confirming our expectation that these changes can be decoupled through strong and phylogenetically non-random range shifts. Going even further, our results showed that local species loss versus gain were strongly phylogenetically selective into opposite directions, especially in the Americas, Europe and Africa. In such assemblages, the net change in SR and in either phylogenetic metric might be very low, but the underlying changes in species composition are nonetheless high, indicating greater shifts in assemblage composition than would be apparent if projected gains and losses of species were not separately assessed. We conclude that disentangling the impacts of species gains and losses can aid in understanding of how assemblage-level phylogenetic diversity might be impacted by climate change, and with it, presumably, the diversity in functional traits and possibly ecosystem functioning. Important aspects of these impacts might be masked if only considering overall change in SR or phylogenetic diversity, or if only focusing on projected species losses.

The projected changes in Faith PD and MPD differ significantly from expectations if random species are being gained or lost across large areas of the globe, indicating that the current phylogenetic structure of individual species assemblages might be widely changed in the future. This finding is in contrast with an earlier study that projected extinction risk from climate change to be evenly distributed across the phylogeny for some taxa within Europe [37]. Differences to large scale studies are probably caused by our focus on species composition in local (spatially explicit) assemblages. However, our results also differ from another regional study, which found mostly random declines in Faith PD under climate change in various mammal, plant and insect families in the Cape Floristic Region of South Africa [19]. This difference in results might reflect variation between taxa or in taxonomic level of analysis, or be due to the other study's focus on a biodiversity hotspot where endemic old lineages exhibit high local species overlap, so that local species range shifts are unlikely to remove entire lineages from the phylogeny.

### (a) Projected spatial patterns of phylogenetic assemblage turnover

As expected, our results show that the spatial patterns of proportional changes in SR and amount of evolutionary history (Faith PD) are highly correlated on a global scale, thus the projected losses as well as gains in assemblage SR are largely reflected in decreases and increases in Faith PD, depending on the phylogenetic tree structure [38,39]. By contrast, the changes in average relatedness (inverse of MPD) are independent from the changes in SR and Faith PD and show an opposite spatial pattern in nearly half of the assemblages, as for example across vast areas of Europe (see electronic supplementary material, discussion). These striking differences are corroborated by the spatial patterns in those changes that are significantly non-random, which also differ strongly between Faith PD and MPD.

We projected a clear spatial pattern where northern latitudes in North America and Eurasia were dominated by increasing phylogenetic clustering or diversification as a consequence of projected net gains in SR and accompanying changes in species composition. Across many assemblages in the high northern latitudes, net increases in evolutionary history were projected to be driven by significant Faith PD decreases caused by species losses on the one hand and less increase than expected under random species gain on the other hand. The resulting changes in species composition indicate the loss of some phylogenetically distinct species in combination with predominant gains of phylogenetically indistinct species. This result is not surprising as high northern latitudes are known to host fewer old lineages [40], but disentangling effects of species loss and gain reveals surprisingly strong projected assemblage-level turnover.

In addition, projected phylogenetic clustering in many of those areas indicated that the gained species are mostly related to each other or to the species already occurring in the assemblage, as shown by significant overall increases in relatedness in North America and patchy areas in northern Asia, for example. This projected combination of local gains of phylogenetically indistinct and closely related species with local loss of phylogenetically distinct and distantly related species is worrying if phylogenetically distinct species also exhibit unique features or traits [41], which might cause them to play unique roles in the functioning of ecosystems. If this is the case, our results indicate that uniform climate-driven range shifts across bird species towards higher latitudes and elevations, which are already observable [6,42,43], might select for species with similar traits in northern latitudes with potential consequences for ecosystem functions (although links among phylogenetic and functional diversity are strongly debated, see [17]). However, many northern areas are projected to experience patterns other than phylogenetic clustering, ameliorating these inferences (e.g. we project increasing phylogenetic diversification in south-central Canada and central Siberia, caused by a net species loss, significant increases in evolutionary history, and significant decreases in relatedness). Again, disentangling these changes from species loss versus gain showed considerable turnover in phylogenetic composition for these assemblages, which might modify ecosystem functioning in unpredictable ways [10,44].

The pattern of phylogenetic homogenization, caused by coinciding projected decreases in evolutionary history and increases in relatedness, occurred least frequently globally, in assemblages which were generally characterized by relatively small absolute changes in all three metrics (SR, Faith PD and MPD; notable in northern latitudes in eastern Europe, Kazakhstan steppes and across the USA). This was somewhat surprising as homogenization is often observed following anthropogenic disturbance, like habitat conversion through urbanization or agricultural expansion or intensification [45,46]. Potentially, these impacts are not drastic at our large spatial grain and extent due to the high mobility of many bird species, and because we modelled range shifts purely based on climate change rather than land-use change projections [26]. Investigating the impacts for other, less mobile taxa or assuming lower dispersal abilities in the models would probably increase the number of assemblages in this group due to potentially higher local extinctions [47].

In contrast with the patterns in northern Eurasia and North America, projected increasing phylogenetic over-dispersion dominated the remaining continents with the exception of Australia, indicating decreases in both evolutionary history and relatedness with fewer species in future assemblages that are very distantly related. Assemblage change in these areas was generally driven by stronger decrease in evolutionary diversity than expected from species loss, and somewhat weaker effects from species gain that significantly increased relatedness. This indicated that across the world and especially in South America and southern Africa, projected local species losses predominate and remove phylogenetically distinct species, whereas projected species gains are less pronounced and add closely related species. These projected patterns indicate widespread local loss of old lineages especially in the southern hemisphere [25,40], and ensuing loss of valuable phylogenetic diversity and unique evolutionary history. Again, if trait diversity is linked to phylogenetic diversity, these results could imply potential widespread functional impoverishment of local assemblages [48], although we show that the underlying mechanism is different compared to the high northern latitudes.

### (b) Data limitations and model uncertainties

Although there are several well-known sources of uncertainty inherent to SDMs, [49], we found largely consistent results across different climate models, model algorithms and dispersal scenarios. Still, projected species distributions as used here to assess the potential changes in all three diversity metrics need to be interpreted with care unless rigorously validated. We were unable to explicitly test the transferability of the model based on current and historic distributions, as such data are unavailable for the vast majority of species. A much-needed validation study for the most well-known historic species distributions could confirm the general applicability of SDMs to projection among time periods. Nevertheless, it would be very difficult to infer how well the models perform for future projections and especially under novel climates. Furthermore, our interpretation of the projected changes in the phylogenetic structure of species assemblages refers to impacts based on ranges shifts under climate change alone, ignoring other drivers such as changes in land-use or biotic interactions, and thus reflects the general trend in which the two phylogenetic diversity metrics could be moving (see supplements for a more detailed description on the study limitations and model uncertainties).

Finally, the phylogeny needs to be interpreted as a hypothesis rather than at face value [16,36], although we could show that phylogenetic uncertainty did not cause strong variation in estimates of phylogenetic diversity. There are also several limitations inherent to macroecological studies at a global scale that are unavoidable due to the scarcity of more highly resolved data. Owing to the coarse resolution of underlying species range maps, the results are robust to outline broad trends in potential assemblage changes but should not be used to assess locality- or species-specific responses.

### (c) Implications for conservation

At a global scale, the preservation of local phylogenetic diversity could be key to the resilience of biodiversity to environmental change [13]. It remains unclear whether assemblages that cover more ancient species and likely higher feature diversity have higher evolutionary potential for adaptation to climate change, or whether those that have undergone recent radiations do [50]. Nevertheless, a high diversity in functional traits has been linked to higher productivity and resilience of ecosystems [51]. Thus, the preservation or even increase of phylogenetic and potentially trait diversity in local species assemblages might turn out to be an advantage when facing environmental change. Our results show strong phylogenetic restructuring across the globe through local species range shifts projected under climate change for the year 2080, which could lead to strong shifts in the functional traits present in local assemblages. We suggest that future ecosystem functioning might be strongly affected in particular by widespread gains of phylogenetically indistinct species (strongest in the northern latitudes) and by some serious losses of phylogenetically distinct species (particularly in the southern hemisphere). Although climate change vulnerability might not be clustered on the global avian phylogeny, our results show the potential for strong heterogeneity in assemblage-level changes that are phylogenetically selective in many regions and remain hidden when only considering net richness changes. Our results therefore reinforce the utility of phylogenetic indices for conservation and of disentangling species losses versus gains for climate impact studies.

## Data Availability

The BirdLife range maps are publicy available [27]. ISIMIP climate data are available from the ISIMIP node of the ESG server (https://esg.pikpotsdam.de/search/isimip/?product=input). The modelled species distributions are publicly available as part of the Terrestrial Biodiversity Sector of ISIMIP (y). The Phylogenetic tree is available (S63). The R code to re-produce the analysis, the species presence absence matrixes needed to re-run the analysis and the result files needed to re-draw the plots are also available on Dryad (doi:10.5061/dryad.cjsxksn6j) [52]. The data are provided in electronic supplementary material [53].
